# Fecal calprotectin and other biomarkers are not prospectively associated with food protein-induced allergic proctocolitis

**DOI:** 10.1002/jpn3.70257

**Published:** 2025-11-11

**Authors:** Timothy Sun, Yamini V. Virkud, Mary Kirpas, Kate Gregory, Jocelyn De Paz, Isabel O’Connell, Moran Yassour, Wayne Shreffler, Qian Yuan, Victoria Martin

**Affiliations:** 1 Pediatric Allergy Immunology, Harvard Medical School, Massachusetts General Hospital, Boston, Massachusetts, USA; 2 Pediatric Allergy Immunology, University of North Carolina, Chapel Hill, North Carolina, USA; 3 Microbiology & Molecular Genetics Department, Faculty of Medicine, The Hebrew University of Jerusalem, Jerusalem, Israel; 4 The Rachel and Selim Benin School of Computer Science and Engineering, The Hebrew University of Jerusalem, Jerusalem, Israel; 5 Pediatric Gastroenterology, Harvard Medical School, Massachusetts General Hospital, Boston, Massachusetts, USA

**Keywords:** 16S rRNA microbiome, eosinophil-derived neurotoxin, reference ranges, Zonulin

## Abstract

**Objectives:**

Diagnosis of food protein-induced allergic proctocolitis (FPIAP) is challenging due to the lack of noninvasive biomarkers. We evaluated fecal calprotectin, eosinophil-derived neurotoxin (EDN), and zonulin as potential biomarkers for diagnosing FPIAP, while also examining normal ranges in healthy infants under 12 months.

**Methods:**

We analyzed 214 stool samples from 115 infants (63 with clinically diagnosed FPIAP) over the first year of life from a large prospective observational cohort. We examined the range for each biomarker in infants over time and compared biomarker concentrations in infants with and without FPIAP using linear mixed-effects modeling. We also compared biomarker concentrations to existing 16S rRNA microbiome profiles using MaAsLin2.

**Results:**

Concentrations of calprotectin, EDN, and zonulin were not associated with FPIAP at the time of diagnosis (*p* = 0.81, *p* = 0.74, *p* = 0.24, nor longitudinally (*p* = 0.356, *p* = 0.0791, *p* = 0.333). Calprotectin was found to decrease significantly over the first 12 months of life for both groups (*p* < 0.001), while EDN and zonulin did not significantly change (*p* = 0.903, *p* = 0.043). There was no associated dysbiosis or microbial signature with any of the three biomarkers.

**Conclusion:**

Concentrations of calprotectin, EDN, and zonulin were not associated with clinically diagnosed FPIAP in our cohort. Very high levels of calprotectin are noted in early infancy in asymptomatic healthy infants, and we provide normal ranges across the first year of life for all three biomarkers. This study does not support the use of fecal calprotectin, EDN, or zonulin for diagnosis of FPIAP.

## Graphical Abstract

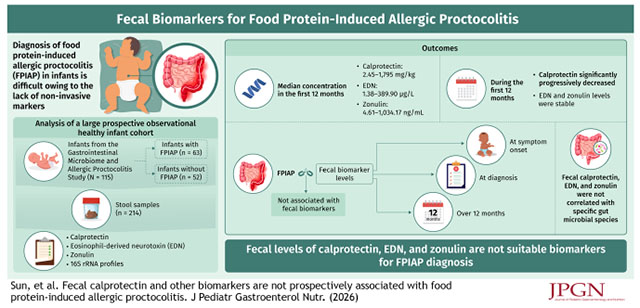

## 1 | INTRODUCTION

Food protein-induced allergic proctocolitis (FPIAP) is a nonimmunoglobulin E (IgE)-mediated food allergy causing diarrhea, bloody and/or mucousy stools, and irritability. Often called cow’s milk protein allergy (CMPA) or milk soy protein intolerance (MSPI), this clinical entity is defined by many nonspecific symptoms and can be difficult to definitively diagnose.^1^ Many infants are described as fussy, have symptoms of reflux, or have growth failure, while others are described as happy and thriving despite bloody stools. It often presents in the first 1–2 months of life and resolves with elimination of the offending food antigen, most commonly cow’s milk protein.^2^ Stool guaiac testing is often used to help with diagnosis, though it has not been validated for this purpose, and rates as high as 30% in healthy infants have been reported.^3,4^ Published guidelines recommend a confirmatory challenge 1 month after symptom resolution for formal diagnosis, but this is often not being done in clinical practice.^3,5–8^

There has been some investigation into stool biomarkers of FPIAP to improve diagnosis, but results have been inconclusive and complicated by differing diagnostic criteria.^9–13^ Previously examined stool biomarkers include zonulin, calprotectin, eosinophil-derived neurotoxin (EDN), tumor necrosis factor-alpha (TNF-a), and more.^9,11,14^ The literature also lacks robust healthy reference ranges for these biomarkers in this very young age group.^11,15,16^ We sought to utilize our large prospective observational healthy infant cohort (the gastrointestinal microbiome and allergic proctocolitis study, or GMAP^17^) to both establish normal ranges for zonulin, calprotectin, and EDN in asymptomatic healthy infants and to evaluate the potential of these biomarkers to differentiate children clinically diagnosed with FPIAP from those without.

Calprotectin is a calcium and zinc binding protein found mainly in neutrophils which when detected in fecal matter is thought to be the result of neutrophil migration to gastrointestinal tissue due to inflammation.^18^ The role of fecal calprotectin in diagnosis and monitoring of intestinal inflammation in pediatric inflammatory bowel disease is well-described, but data supporting its role in FPIAP is less clear.^9,12,19,20^ The use of proton pump inhibitors (PPIs) is common for children with FPIAP, and it has been shown to elevate fecal calprotectin.^17,21^

Zonulin, a protein that modulates the tight junctions in the jejunum and ileum, has been used as a marker of intestinal permeability. Fecal zonulin, however, has been more variable than serum zonulin.^22^ There is even more limited data for ranges in children under 12 months of age.^9,16^ Two groups found conflicting results regarding directionality of zonulin, despite both using 100 full-term infants from the same city in Poland.^9,23^

EDN is a degranulation product of eosinophils, often involved in allergic and immune responses.^24^ Infants with FPIAP were previously identified to have an eosinophil-predominant histopathology, making EDN a particularly promising potential biomarker for this disease.^25,26^ There is minimal existing literature around the utility of fecal EDN in the diagnosis of FPIAP, as well as similarly limited datasets regarding normal ranges in children under 12 months of age.^11,14,27^

The intestinal microbiome is partially responsible for helping to maintain immune and metabolic homeostasis and evolves and develops rapidly in the first 12 months of age.^28–33^ Prior results from the GMAP cohort showed key microbiome signatures in infants with FPIAP (even before symptom onset) compared to those without.^31^ One group found calprotectin to associate with certain *Enterocloster* and *Clostridium* species at 6 months stratifying by delivery mode.^32^ Other groups have found that dysbiosis can correlate with fecal biomarkers.^34^ We therefore also sought to evaluate potential associations between strong biomarker signals and particular taxonomic features or signatures in the intestinal microbiome using Shannon diversity index. This index is useful in measuring gut microbiota diversity where a higher diversity is generally considered normal.

## 2 | METHODS

### 2.1 | Ethics statement

The GMAP Study was approved by the Massachusetts General Hospital Institutional Review Board (IRB) and a parent of all enrolled infants gave written informed consent.

### 2.2 | Sample selection and processing

Infants were selected from the GMAP cohort, a prospective observational study, which enrolled healthy infants in suburban Massachusetts at their first well visit starting in March 2014 and will be following them through 18 years of age.^17^ Infants with FPIAP were defined by prespecified case inclusion criteria (including guaiac positive or grossly bloody stool, pediatrician diagnosis, and study staff chart review).^17^ Pediatrician diagnosis meant that they documented the diagnosis in the chart and or in our prospectively administered case report forms as part of the prospective study, had symptoms consistent with disease in study staff chart review, and had either guaiac positive or grossly bloody stools. We noted cases that were diagnosed based on less stringent criteria such as no blood in the stool or less clear symptoms but those were eliminated from analyses as outlined in our primary outcomes paper.^17^ Charts of infants were reviewed by study staff for evidence of exposure to PPI and length of therapy was extracted when available. Stool samples were collected from all participants at every well-child visit as previously published.^31^ For these analyses, we selected 214 samples from 115 infants (63 with clinically diagnosed FPIAP, 52 without) with adequate sample material, including longitudinal sampling when available, using a nested case-control design similar to previously published.^31^ Biomarker data were generated in 2022, and integrated analyses were done in 2023–2024, incorporating the microbiome data. Samples for zonulin were extracted using the IDK Zonulin ELISA kit protocol to obtain sample concentrations. Samples for EDN and calprotectin were extracted in the EliA Calprotectin 2 Extraction buffer using the EliA Stool Extraction kit 2. EDN was processed using the ImmunoCAP EDN Assay Kit, while calprotectin was processed using the EliA Calprotectin 2 protocol, then concentrations for both were measured on the Phadia 250 Laboratory System. 16S rRNA sequencing and analysis methods have been previously published.^31^

### 2.3 | Statistical analysis

Samples were plotted for outliers and significantly elevated values were removed from analysis if they were greater than four standard deviations from the median (*n* = 1). Samples measuring out of range for the relevant assay were repeated when sample allowed or removed from analyses if consumed. Reference ranges for calprotectin values are binned in 4-month intervals for better precision due to the wide range over different ages. EDN and zonulin are binned as one group due to the smaller range. For all biomarkers, we provide the median, 75th, and 95th percentile values for 0–12 months. Concentration was plotted against time (ggplot) and linear mixed effects models (lmer) were used to examine each biomarker over time in infants with FPIAP compared to those without with significance set at *α* < 0.01, using R Version 4.3.0 (packages: tidyverse 2.0.0, tableone 0.13.2, lmerTest_3.1-3). A series of models was constructed using random effects for subjects, and fixed effects for FPIAP and age at sampling. The concentration of each biomarker was plotted against age, FPIAP diagnosis, and other metadata. Wilcoxon rank sum was used to compare samples at the time of diagnosis to age-matched controls. Shannon diversity index was calculated for infants with available 16S microbiome data collected as previously published,^31^ and associations with any biomarkers were evaluated using Maaslin2. A summary of models constructed, and tests/equations used is shown in Table S1. We had 80% power with an alpha 0.05 to detect a 200 mg/kg difference in calprotectin.

## 3 | RESULTS

A sample map detailing sampling times for the 214 samples from 115 infants is shown in Figure S1, with demographics similar to the parent cohort shown in Table S2.

### 3.1 | Healthy ranges of fecal calprotectin, EDN, and zonulin in the first 12 months

**FIGURE 1:**
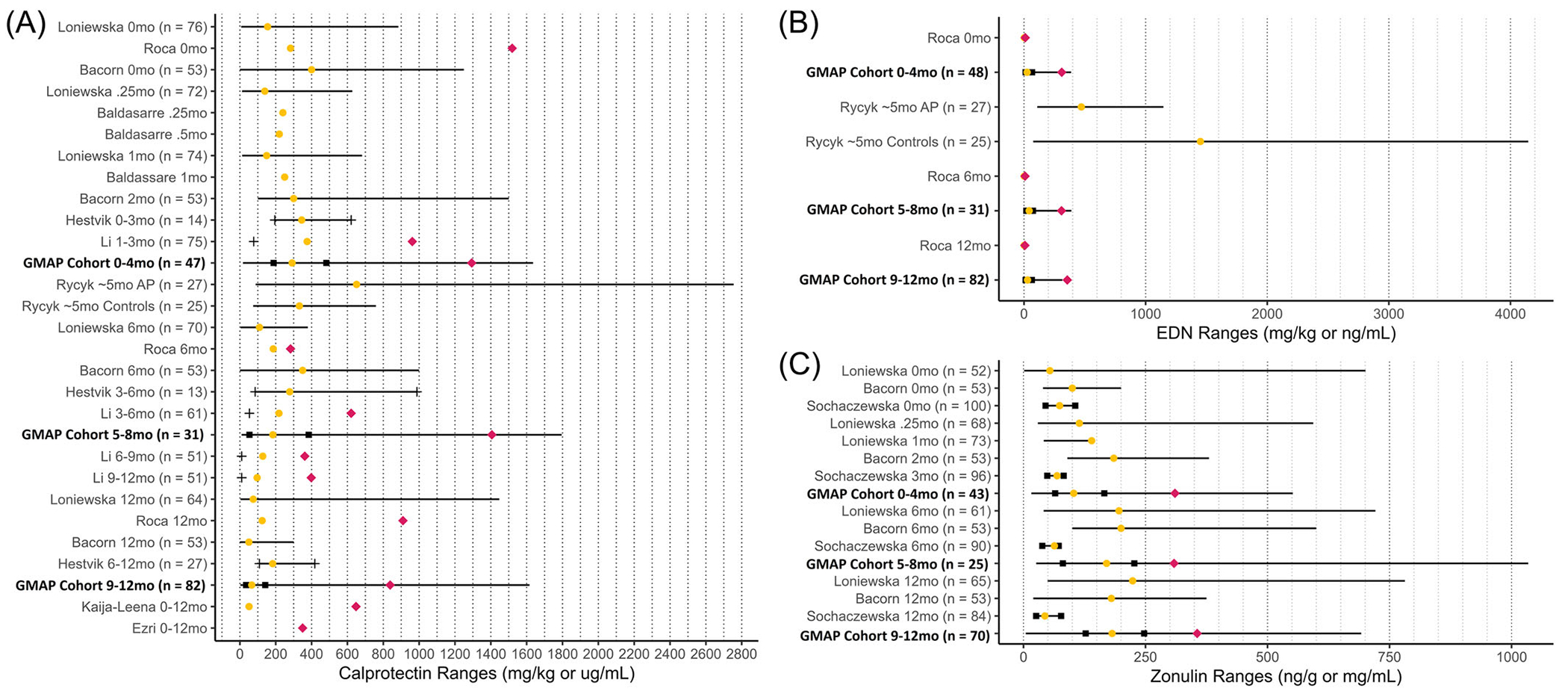
Summary of existing reference ranges for biomarkers in the literature, with new data from our cohort highlighted in bold. Sample sizes are included where possible. (A) Fecal calprotectin values. Of note, Łoniewska et al. reported results in μg/mL while the other groups used mg/kg. (B) Fecal zonulin values. Of note, Sochaczewska et al. reported results in ng/g while all other groups reported mg/mL. (C) Fecal eosinophil-derived neurotoxin values. Rycyk et al. reported in ng/mL while Roca et al. and our group reported in mg/kg.

Fecal calprotectin concentrations decreased significantly over time (*p* < 0.001), with very high levels in the earliest months, while levels of EDN and zonulin were static longitudinally over the first 12 months of age (*p* = 0.903, *p* = 0.043 respectively). Over the first 12 months, median calprotectin was 143.0 mg/kg with (2.45–1795) mg/kg range, median EDN concentration was 29.45 μg/L with (1.38–389.90) μg/L range, and median zonulin concentration was 153.27 ng/mL with (4.61–1034.17) ng/mL range. Table 1 provides reference ranges for the three biomarkers of interest from all analyzed samples. A more detailed version can be found in Table S3. Our results plotted alongside previously reported values in the existing literature are shown in Figure 1. Of note, there was no association between any of these three biomarkers and mode of delivery, exposure to antibiotics during delivery, perinatal antibiotic exposure, diet at birth, atopic dermatitis status, or IgE-mediated food allergy status (data not shown). Calprotectin was also not significantly associated with diet type at the time of diagnosis, whether or not the child has grossly bloody stool, nor which treatment strategy resulted in symptom resolution (maternal diet change, infant formula change, maternal diet and infant formula change, or only infant probiotic use).

### 3.2 | Differences in fecal biomarkers are not associated with for the diagnosis of FPIAP

**FIGURE 2:**
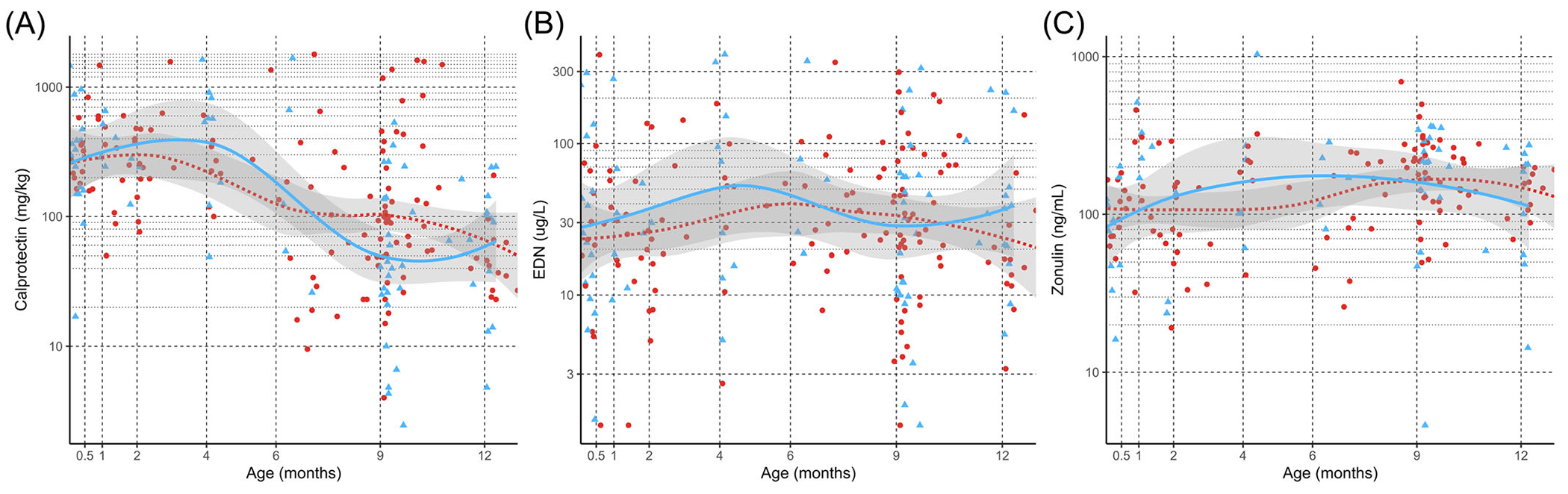
Longitudinal analyses of biomarker concentrations over the first 12 months in infants with and without FPIAP. (A) Calprotectin decreases significantly over the first 12 months of life but does not differentiate between infants with and without FPIAP. (B) eosinophil-derived neurotoxin and (C) Zonulin do not significantly change over time nor significantly differentiate between infants with or without FPIAP. FPIAP, food protein-induced allergic proctocolitis.

**FIGURE 3:**
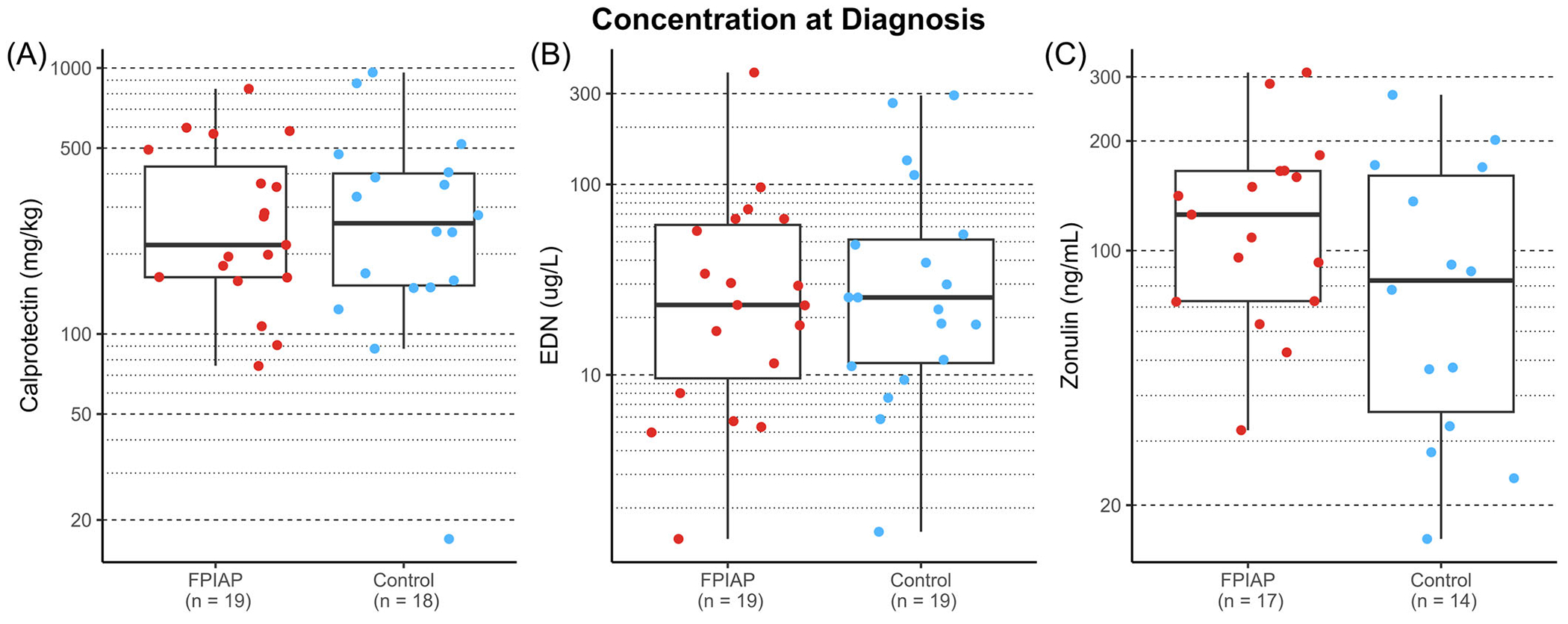
There is no difference between levels of (A) calprotectin, (B) eosinophil-derived neurotoxin, and (C) zonulin, at the time of diagnosis of FPIAP compared to age-matched asymptomatic controls. FPIAP, food protein-induced allergic proctocolitis.

We found no significant differences in calprotectin, EDN, or zonulin concentrations longitudinally across the first 12 months between infants with FPIAP and those without (Figure 2; *p* = 0.356, *p* = 0.0791, *p* = 0.333, respectively). These results were not changed by including PPI use in the model. We also examined each biomarker at the time of diagnosis of FPIAP when intestinal inflammation would be expected to be highest, compared to age-matched controls, and we still did not find any statistically significant differences between diagnosis time and calprotectin, EDN, or zonulin (Figure 3; *p* = 0.81, *p* = 0.74, *p* = 0.24, respectively). Separately of FPIAP diagnosis, PPIs, but not H2 blockers, were associated with elevated fecal calprotectin levels. Sixteen samples from seven infants who were exposed to PPI therapy had increased levels of fecal calprotectin (Figure 2, *p* < 0.001).

Similarly, there were no differences in any biomarkers in infants with FPIAP from sample(s) during symptom onset (before the diagnosis was made) compared to age-matched controls (Figure S3; *p* = 0.90, *p* = 0.51, *p* = 0.40 for calprotectin, EDN, and zonulin, respectively).

### 3.3 | Associations with fecal calprotectin, EDN, zonulin, and the microbiome

We analyzed the biomarker trajectories to examine whether there were associations between biomarkers levels and specific microbiome signatures or taxa. We found a positive association between the Shannon diversity index of the microbial community and the concentration of calprotectin (Figure S4, *p* = 0.002) which was likely confounded by age. No significance was found between microbiome diversity and zonulin or EDN (*p* = 0.280 and 0.736 respectively). No particular species tracked with any of the biomarkers using a q-value of 0.2 and an absolute coefficient of <0.01 and PPI usage was not associated with Shannon index.

## 4 | DISCUSSION

To our knowledge, this is the largest longitudinal fecal biomarker study conducted on infants under 12 months old designed to assess for associations with FPIAP. We provide reference ranges for infants under 12 months of age for fecal calprotectin, zonulin, and EDN. Fecal calprotectin is notably high in early infancy and decreases significantly over the first 12 months. We did not find any significant differences (longitudinally or at the time of symptom onset or diagnosis) between infants with FPIAP and unaffected controls in levels of EDN, calprotectin, or zonulin in the stool. Of note, PPI use was associated with an increased calprotectin level, which has been previously reported in other patient populations.^21^

Calprotectin is one of the most widely studied stool biomarkers in children, particularly in children with inflammatory bowel disease, but the range is very wide in early infancy and values in the thousands have been frequently reported in healthy young infants. There are several proposed explanations for this, including immaturity and permeability of infant digestive tracts.^28–30^ Our study agrees with prior work and highlights the importance of careful interpretations of elevated calprotectin in early infancy and references ranges in small age bins are provided. Importantly, there was no association between a clinical diagnosis of FPIAP and level of fecal calprotectin in this large well-characterized cohort, and so we wouldn’t recommend sending or using it for this diagnostic question. This is consistent with a few smaller studies on FPIAP.^9,12^ Rycyk et al. observed significantly higher calprotectin and EDN in children with FPIAP than children with functional gastrointestinal disorders, but was limited to one time point with a limited study size.^11^ There is even more limited and conflicting literature examining levels of zonulin and EDN in FPIAP but we did not find these biomarkers helpful in discriminating infants with and without FPIAP.^9,11,23,27^

In agreement with existing literature,^21^ we found significantly higher levels of fecal calprotectin in children being treated with PPIs. The mechanism of this isn’t well understood; but it has been suggested that the lowered acidity from PPIs supports bacterial overgrowth, stimulating neutrophil migration.^35–37^ We compared the biomarker levels with our pre-existing 16S microbiome data and did not find any association between microbial signatures or dysbiosis and any of the three biomarkers. Regardless, this effect should be considered when interpreting calprotectin results in children on PPIs.

Our cohort’s strength lies in its size, longitudinal sampling, comprehensive clinical metadata, and well-characterized population of infants diagnosed with FPIAP. Cohort limitations include lack of racial diversity as previously reported.^17^ Limitations of this nested case-control biomarker study include a limited number of available longitudinal samples from a given patient. Given the observational design of the cohort at large, infants were diagnosed clinically with FPIAP (as is done most commonly in clinical practice in the United States) meaning these biomarkers could not be compared against a diagnosis made by oral food challenges or rectal biopsies. Future studies aimed at better endophenotyping of infants with this range of non-IgE-mediated cow’s milk protein allergies are needed.

## 5 | CONCLUSION

In conclusion, fecal calprotectin, EDN, and zonulin levels were not associated with a clinical diagnosis of FPIAP in our cohort. Therefore, we would not recommend sending these biomarkers to aid clinical decision-making. We provide reference ranges for all three biomarkers in the context of previous literature, to aid in interpretation of these biomarkers in infants under 12 months of age, particularly in the earliest months. Calprotectin is very high in many healthy infants early in infancy and decreases significantly over the first 12 months of age, so interpretation of high levels in very young infants is challenging and/or unlikely to differentiate intestinal health from disease. We recommend all clinicians follow published international guidelines^5–7^ for diagnosis of FPIAP/CMPA/non-IgE-mediated CMA using an open milk protein challenge 1 month after symptom resolution on a diet change. It is important to make this diagnosis systematically and to avoid overdiagnosis or unnecessary long-term allergen avoidance (which may precipitate more life-threatening IgE-mediated allergy). Future studies including diagnostic challenges may improve our ability to identify noninvasive biomarkers, by improving heterogeneity in diagnosed study populations.

## Supplementary Material

Sup Fig 3

Sup Fig 1

Sup Fig 2

Sup Fig 4

Sup Tab 1

Sup Tab 2

Sup Tab 3

Additional supporting information can be found online in the Supporting Information section at the end of this article.

## Figures and Tables

**TABLE 1:** Ranges of values for all infants sampled.

Calprotectin (mg/kg)				Zonulin (ng/mL)			
Age group	Median	75th %ile	95th %ile	Age group	Median	75th %ile	95th %ile
0–4 months (59)	291.5	482.5	1293.5	0–12 months (178)	153.27	223.65	344.37
5–8 months (29)	184.0	383.5	1405.9	EDN (μg/L)			
9–12 months (90)	66.0	141.5	838.2	0–12 months (214)	29.5	66.0	225.65

*Note*: Zonulin and EDN are presented as one age range 0–12 as no significant changes over time were observed. Number of samples per group is written in parentheses.

Abbreviation: EDN, eosinophil-derived neurotoxin.

## What is Known

- Fecal calprotectin is a biomarker of inflammation in the gut and has been used to help diagnose inflammatory bowel conditions.
- Fecal calprotectin of over 50 mg/kg is considered elevated in adults.
- Fecal calprotectin is elevated in infants and decreases in the first few months of life.

## What is New

- In our cohort, fecal calprotectin, eosinophil-derived neurotoxin, and zonulin did not distinguish those with or without food protein-induced allergic proctocolitis, and therefore do not appear to be useful in the diagnosis or management of food protein induced allergic proctocolitis.
- This study provides reference data from the largest US-based prospective cohort study to establish normal ranges for infants for fecal calprotectin, eosinophil-derived neurotoxin, and zonulin.
