# Supplementary figures and images for: Fecal calprotectin and other biomarkers are not prospectively associated with food protein-induced allergic proctocolitis
Source: J Pediatr Gastroenterol Nutr. Author manuscript; Available in PMC 2026 Apr 2. (PMC13044856; doi:10.1002/jpn3.70257)

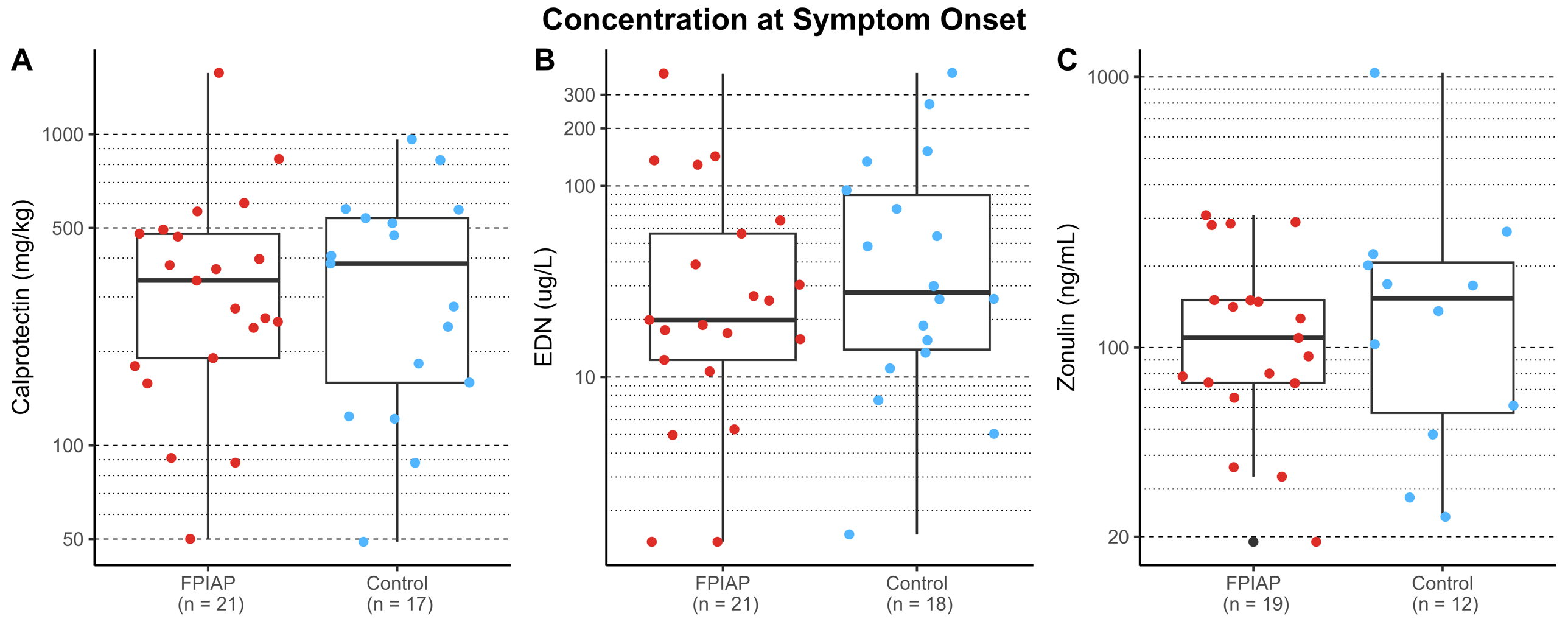

Supplement: Sup Fig 3 [file NIHMS2155150-supplement-Sup_Fig_3.png]

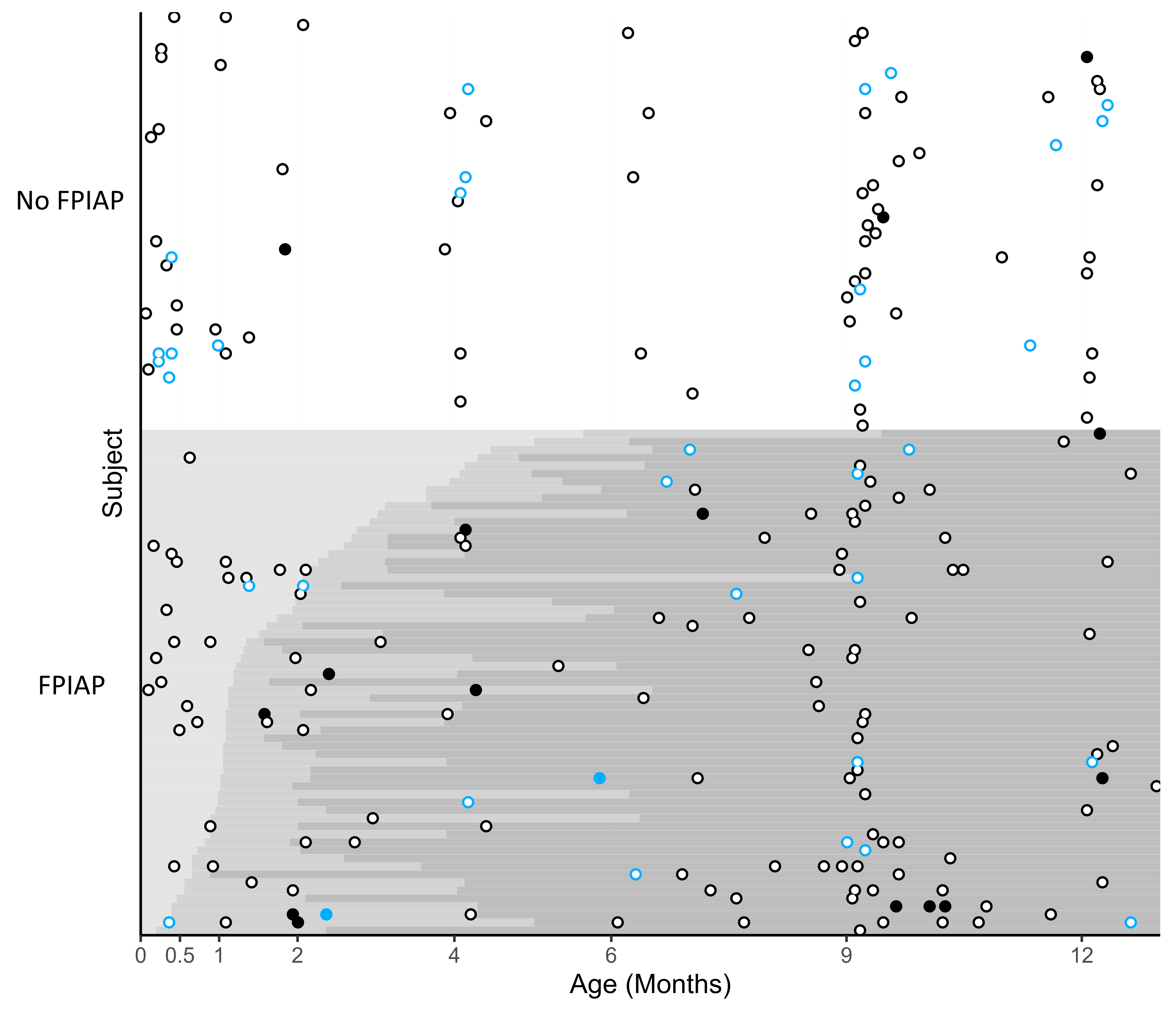

Supplement: Sup Fig 1 [file NIHMS2155150-supplement-Sup_Fig_1.png]

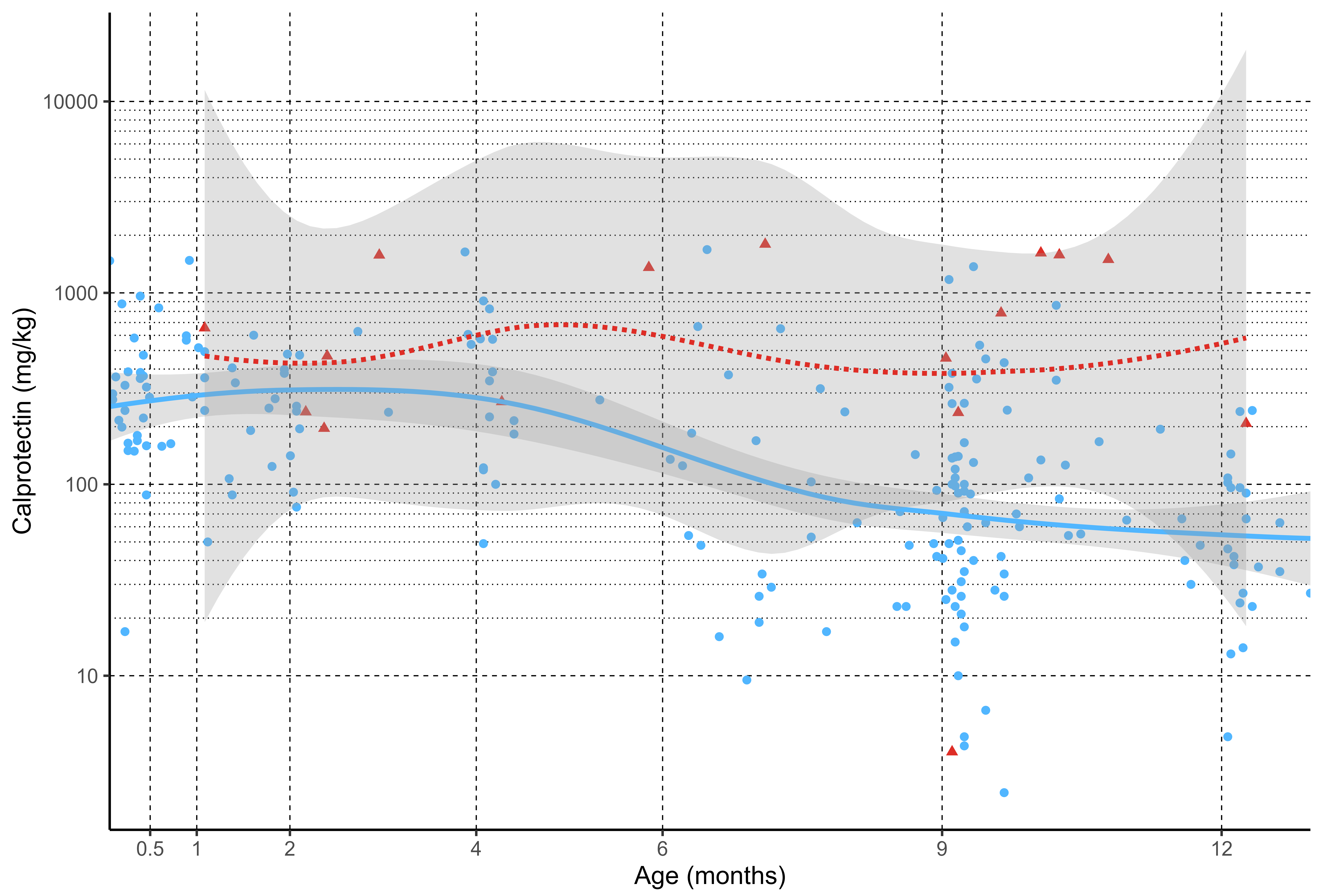

Supplement: Sup Fig 2 [file NIHMS2155150-supplement-Sup_Fig_2.png]

Supplemental Figure 4 Legend.


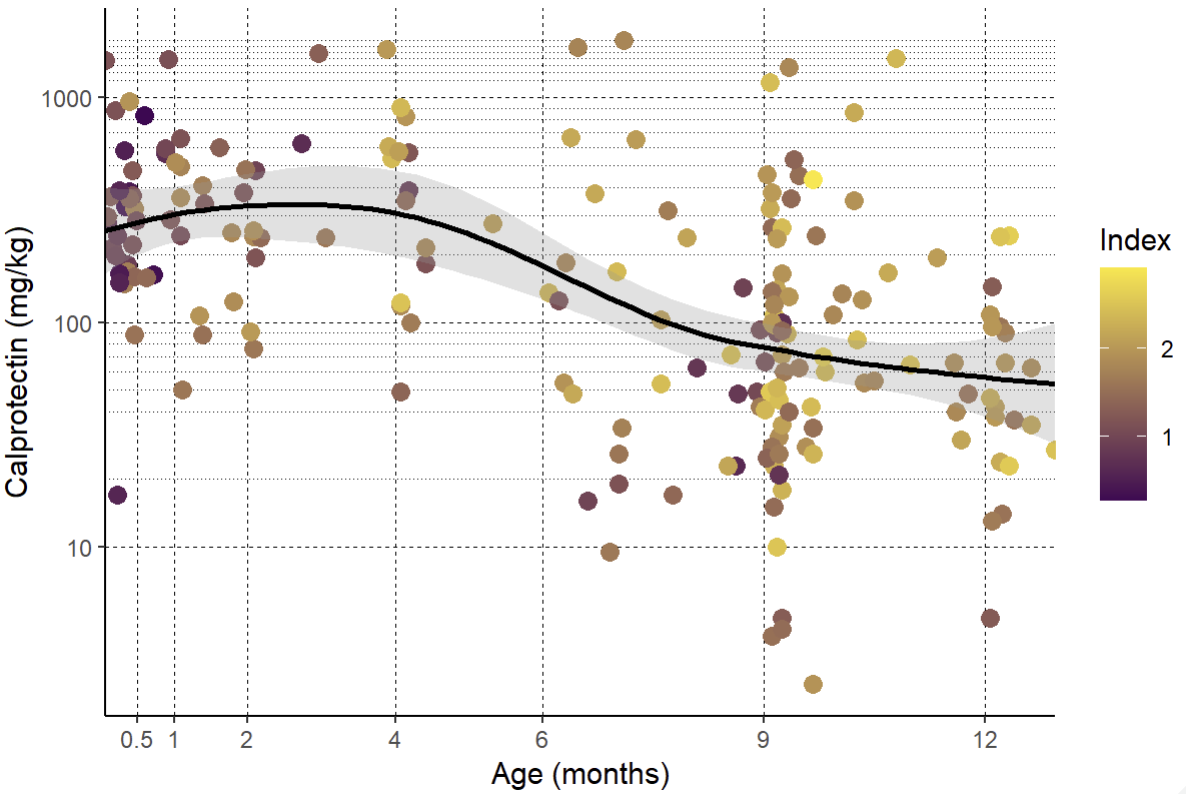

Supplement: Sup Fig 4 [file NIHMS2155150-supplement-Sup_Fig_4.docx]

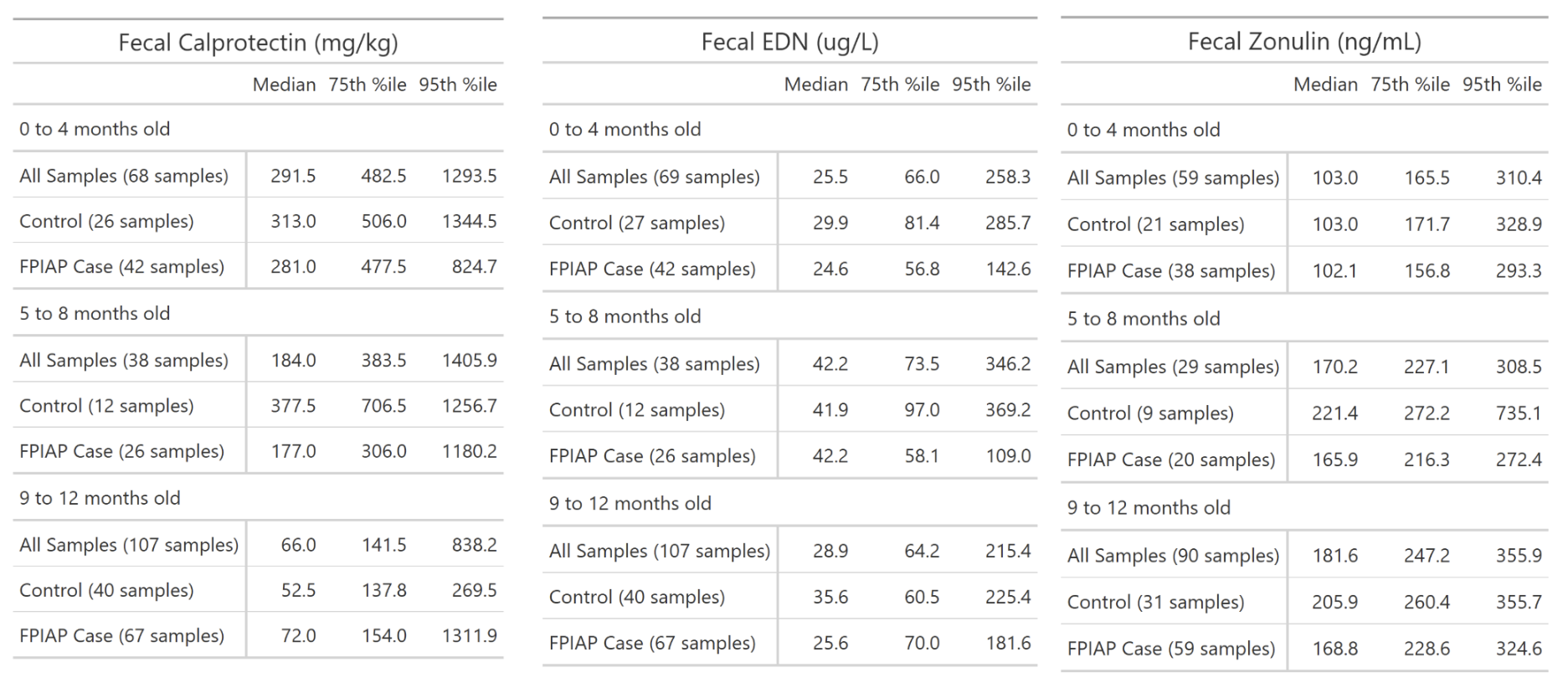

Supplement: Sup Tab 3 [file NIHMS2155150-supplement-Sup_Tab_3.docx]
