# Supplementary material for: Fecal calprotectin and other biomarkers are not prospectively associated with food protein-induced allergic proctocolitis
Source: J Pediatr Gastroenterol Nutr. Author manuscript; Available in PMC 2026 Apr 2. (PMC13044856; doi:10.1002/jpn3.70257)
Supplement: Sup Tab 1 [file NIHMS2155150-supplement-Sup_Tab_1.docx]

| Endpoints | Methods | Variables |
| --- | --- | --- |
| (1) Association between biomarker concentration of subjects with age  (2) Association between biomarker concentration and FPIAP status, adjusted for age  (3) Association between PPI usage and calprotectin concentration  (4) Association between biomarker concentration and Shannon index  *Biomarker variable includes calprotectin, zonulin, and EDN concentrations* | Linear Mixed Effects Model  **y** = **Xβ** + **Ζu** + **ε** | **y** = continuous variable of biomarker concentration  **Ζ** = Subject-specific random effects  (1) **X** = age (continuous)  (2) **X** = FPIAP status (binary), age (continuous)  (3) **X** = PPI usage (binary), age (continuous)  (4) **X** = index (continuous), age (continuous),  *Age, PPI usage, index are time varying* |
|  |  |  |
| Comparison between biomarker concentration of subjects with FPIAP at time of diagnosis and age matched controls | Wilcoxon Rank Sum | Comparison Groups:  FPIAP Status (FPIAP vs No FPIAP) |
