# Supplementary material for: Fecal calprotectin and other biomarkers are not prospectively associated with food protein-induced allergic proctocolitis
Source: J Pediatr Gastroenterol Nutr. Author manuscript; Available in PMC 2026 Apr 2. (PMC13044856; doi:10.1002/jpn3.70257)
Supplement: Sup Tab 2 [file NIHMS2155150-supplement-Sup_Tab_2.docx]

|  | FPIAP | Control |
| --- | --- | --- |
| N | 63 | 52 |
| Female | 28 (44.4%) | 21 (40.4%) |
| Race |  |  |
| White | 42 (67.7%) | 30 (58.8%) |
| Black | 1 (1.6%) | 1 (2.0%) |
| Asian | 14 (22.6%) | 11 (21.6%) |
| Other | 1 (1.6%) | 1 (2.0%) |
| Multiple Race | 4 (6.5%) | 8 (15.7%) |
| Not Hispanic or Latino | 47 (90.4%) | 39 (97.5%) |
| C-section | 22 (34.9%) | 17 (32.7%) |
| Antibiotics During Delivery | 32 (50.8%) | 27 (51.9%) |
| Initial Diet |  |  |
| Exclusively Breastfed | 41 (65.1%) | 30 (57.7%) |
| Exclusively Formula | 7 (11.1%) | 5 (9.6%) |
| Partially Breastfed | 15 (23.8%) | 17 (32.7%) |
| IgE-mediated Food Allergy | 8 (12.7%) | 0 (0.0%) |
| Eczema | 35 (55.6%) | 22 (42.3%) |
